# Ghrelin Modulates Differential Expression of Genes Relevant to Immune Activities and Antimicrobial Peptides in Primary Head Kidney Cells of Rainbow Trout (*Oncorhynchus mykiss*)

**DOI:** 10.3390/ani13101683

**Published:** 2023-05-18

**Authors:** Yueh-Chiang Han, Douglas W. Leaman, Brian S. Shepherd

**Affiliations:** 1 ORISE/ORAU/USDA-ARS, School of Freshwater Sciences, University of Wisconsin-Milwaukee, Milwaukee, WI 53204, USA; 2 College of Sciences, Auburn University at Montgomery, Montgomery, AL 36117, USA; dleaman@aum.edu; 3 USDA-ARS, School of Freshwater Sciences, University of Wisconsin-Milwaukee, Milwaukee, WI 53204, USA; brian.shepherd@usda.gov

**Keywords:** ghrelin, differential expression, primary head kidney cells, immunomodulation, antimicrobial peptide, rainbow trout

## Abstract

**Simple Summary:**

Ghrelin is a peptide hormone that regulates food intake and energy balance, but also acts as a cytokine in many species to modulate various immune activities. Multiple ghrelin isoforms were discovered in rainbow trout, but the roles of most isoforms are undefined. This study examined the modulation of immune genes by two major ghrelin variants; the originally identified isoform and its three amino acids truncated isoform. After treating primary cells from rainbow trout head kidney with the two ghrelin analogs, the expression patterns of selected immune-related genes were profiled over time by reverse transcriptase-coupled qPCR. The results suggest that the two ghrelin isoforms exerted divergent modulatory effects on various genes at different time points. These results provide further insight into the direct actions of ghrelin as a critical component of the rainbow trout immune system.

**Abstract:**

Ghrelin is a peptide hormone/cytokine that regulates metabolic processes and plays essential roles in the immune system. To evaluate the immunomodulatory actions of ghrelin isoforms in rainbow trout (RT), an in vitro model was utilized with primary cells isolated from fish head kidney (HKD). These RT-HKD cells were treated with synthetic rainbow trout ghrelin and its truncated isoform, desVRQ-ghrelin, over time (0, 2, 4, and 24 h). Reverse transcriptase-coupled qPCR was used to measure the differential expression patterns of genes relevant to various immune processes and genes of antimicrobial peptides. Ghrelin isoform treatments resulted in functional perturbations that displayed overlapping and divergent patterns of gene expression. The differing actions between the two ghrelin isoforms on various assessed genes, and at differing time points, suggested that the two analogs may activate unique pathways, thereby eliciting distinct responses in fish immunity.

## 1. Introduction

The regulation of innate and adaptive immune systems is critical to animal defense against infectious diseases. Ghrelin is an essential, and multifunctional biomolecule that regulates food intake and energy balance [1,2,3], but also modulates immune processes in many animals [4,5,6,7]. This peptide hormone was first identified in rat stomachs as an endogenous ligand that binds a 7-transmembrane G-protein coupled receptor, called growth hormone secretagogue receptor (GHSR), to mediate its biological effects [1]. The initially discovered ghrelin polypeptide consisted of 28 amino acids, with the third serine *O*-octanoylated. This acylation plays a critical role in receptor binding and eliciting biological activity. Ghrelin is synthesized by X/A-like enteroendocrine cells, predominantly in the gastrointestinal tract, but is also found in multiple tissues including digestive organs, the central nervous system, and the immune system [2,8,9]. The ghrelin peptide has been described in many vertebrates including fish, birds, amphibians, reptiles, and mammals [1,10,11,12]. For teleost fish, ghrelin mRNAs were initially cloned, and peptides were purified from multiple species including channel catfish, eel, goldfish, and tilapia [13,14,15,16]. Further work showed that ghrelin peptides were present in the stomach of rainbow trout (*Oncorhynchus mykiss*) and expression of these mRNAs was confirmed in various tissues [13]. The active isoforms of the mature polypeptide are the basic 23 amino acid rainbow trout ghrelin (rt-Ghrl), and a truncated 20 amino acid isoform termed rainbow trout desVRQ ghrelin (rt-desVRQ-Ghrl) that results from alternative splicing of the ghrelin gene [13].

Ghrelin mediates immune responses in a variety of species. In humans, ghrelin inhibits the expression of pro-inflammatory cytokines in T cells and monocytes [17], and exhibits anti-inflammatory effects mediated by the nuclear factor kappa b (NF-κB) pathway in endothelium [18]. Ghrelin stimulates phagocytosis and superoxide production in rainbow trout primary leukocytes [19]. The liver-expressed antimicrobial peptide 2 (LEAP2) can bind with high affinity to Ghsr1a, thus antagonizing the binding of ghrelin to its receptor in an ancient fish (Indian Ocean coelacanth, *Latimeria chalumnae*) [20]. While evidence suggests that ghrelin modulates innate and adaptive immune responses through multiple immune pathways, much remains unclear about how it mediates its modulatory effects in the vertebrate host immune system [21].

In teleost fish, the head kidney is the equivalent of the bone marrow in higher vertebrates [8]. As such, the head kidney is the major site of hematopoietic stem cell production and the primary lymphoid organ, where lymphocytes were produced and maintain various myeloid lineage cells, which are progenitors to the mature mast cell, granulocytes, and monocytes [8,22]. Recent work has underscored the benefits of using teleost primary cells as a direct and effective alternative to live animal testing when evaluating immunomodulatory processes and vaccine efficacy [23]. Against this background, we examined the regulatory effects of rt-Ghrl and rt-desVRQ-Ghrl using primary rainbow trout (RT)-head kidney (HKD) cell culture as an in vitro model. The expression patterns of selected genes of interest (GOI) from several immune-relevant pathways were profiled by real-time quantitative polymerase chain reaction (qPCR). The results of this research can provide further insights into the direct actions of ghrelin as an element of the rainbow trout immune system, which could lead to potential applications for improving rainbow trout immunity in the aquaculture industry.

## 2. Materials and Methods

### 2.1. Synthesis of Ghrelin Analogs

Peptide sequences for the two active isoforms of rainbow trout ghrelin [13], rt-Ghrl (GSSFLSPSQKPQVRQGKGKPPRV) and rt-desVRQ-Ghrl (GSSFLSPSQKPQGKGKPPRV) were submitted to LifeTein, LLC (Somerset, NJ, USA) for synthesis. Both peptides were third serine *O*-octanoic acid acylated and C-terminal amidated. The synthetic peptides were purified via high-performance liquid chromatography, with purity >97%, and confirmed based on their molecular weights via mass spectrometry by the manufacturer.

### 2.2. Fish

The experimental rainbow trout utilized in this study were purchased from Troutlodge, Inc. (Bonney Lake, WA, USA) as bioassay grade fry (Bonney Lake, WA, USA). A total of 124 mature fish (1.5 years old, 1.6 ± 0.11 kg) were cultured, and 36 fish were used for experiment. All fish were maintained in tanks (2.44 m × 1.22 m, 3600 L). Tanks were supplied with continuous flow (50 L/min) of dechlorinated municipal water at the School of Freshwater Sciences at University of Wisconsin, Milwaukee, and photoperiod was maintained at 12 h light:12 h dark. Husbandry conditions were water temperature 11–15 °C, pH 7.6, and dissolved oxygen 9 −11 mg/L. Animals were fed 4.0 mm floating classic trout diet (Skretting, Tooele, UT, USA) to 10% body weight/day dispensed over 4 feedings/day with vibratory feeders (Sweeney, Boerne, TX, USA). Animal and food wastes were directly removed by single flow-through water system, the continuous flow rate (50 L/min) was sufficient to ensure no detectable ammonia, and the water quality was constantly monitored every two weeks by FF-1A Fish Farming Test Kit (Hach, Loveland, CO, USA). Prior to tissue collection of head kidney, fish were anesthetized by immersion in MS-222 (100 mg/L, Tricaine methanesulfonate, Syndel, Ferndale, WA, buffered with 200 mg/L of sodium bicarbonate). Fully anesthetized animals were then fully bled and euthanized by decapitation. All experiments were conducted following approval and guidelines of the Institutional Animal Care and Use Committee (IACUC) of the University of Wisconsin Milwaukee (IACUC-UWM protocol 19-20 #11&12).

### 2.3. Tissue Collection and Cell Culture

Primary cells from rainbow trout head kidneys were isolated by modified method as described [24]. The head kidney tissue (~0.5 inches most anterior of trunk kidney) was removed from euthanized fish and placed into sterile plates with sterile-filtered Dulbecco’s modified eagle medium (DMEM, ThermoFisher, Waltham, MA, USA). Head kidney tissue was minced with sterile forceps in DMEM, and the suspension was passed through 100 μm cell strainers (Greiner Bio-One, Monroe, NC, USA). Remaining tissue/residue on the cell strainer was further ground and pushed through the sterile 100 μm nylon mesh with pestles and washed twice with DMEM. The resulting cell suspension was filtered with 40 μm cell strainer (Greiner Bio-One, Monroe, NC, USA) and the cells were pelleted at 200× *g* for 5 min. The pellet was re-suspended in Leibovitz medium L-15, (Cytiva, Marlborough, MA, USA) supplemented with 100 IU/mL penicillin, 100 μg/mL streptomycin (Gibco, Amarillo, TX, USA) and 10% fetal bovine serum (Hyclone Characterized FBS, gamma irradiated and heat-inactivated, Logan, UT, USA). The primary cells isolated from each head kidney were evenly plated to four T25 culture flasks (Celltreat, Pepperell, MA, USA) with an average seeding density = 5 × 10^6^ cells/mL and cultured at 15 °C for a total of 7 days. The nonadherent cells were removed after the first 24 h, the adherent cells were rinsed with sterile 1× phosphate-buffered saline (PBS) one time, and medium was replaced with fresh medium. Subsequently, the adherent cells were incubated for an additional 6 days with daily removal of nonadherent cells. Images of living culture were captured every day using an EVOS FL microscopy system (ThermoFisher, Waltham, MA, USA) at 100× and 400× magnification to monitor the morphological changes in immune relevant cells, and most of differentiation was achieved at 6-day post removal of non-adherent cells from RT-HKD.

### 2.4. Ghrelin Treatment and RNA Extraction

Prior to ghrelin treatment, the adherent RT-HKD cells were left undisturbed for 24 h in L-15 medium (without any supplement), washed twice with fresh sterile L-15, and treated for 0 h, 2 h, 4 h, and 24 h with 3 mL of L-15 containing rt-Ghrl at a final concentration of 1.25 ng/μL, and rt-desVRQ-Ghrl at final concentration of 1.11 ng/μL. Four replicates of treatments (control, rt-Ghrl, and rt-desVRQ-Ghrl) and sampling time points (0 h, 2 h, 4 h, and 24 h) were randomly assigned to T25 culture flask containing RT-HKD cells from independent fish. In total, 112 living cultures were utilized to cover all experimental conditions. At the end of each treatment, medium was removed, cells were washed once with sterile PBS, and RNAzol RT was added to each flask to lyse cells and obtain total RNA according to the manufacturer’s protocol (Molecular Research Center, Cincinnati, OH, USA). Afterward, the isolated total RNA was treated with DNase, and purified by spin column chromatography (Norgen Biotek Corp., Thorold, ON, Canada). Total RNA was initially quantified by UV spectrophotometry (Nanodrop, ThermoFisher Scientific, Waltham, MA, USA) with high purity (260/280 ratios ≥ 2.0). Prior to first-strand cDNA synthesis, total RNA was further quantified by fluoroscopy with Qubit fluorimeter (Thermo-Fisher Scientific, Waltham, MA, USA). First-strand cDNA was synthesized from each RNA sample (4 μg/40 μL) with the High-Capacity cDNA reverse transcription kit (Applied Biosystems, Waltham, MA, USA) by following the manufacturer’s recommended protocol. The synthetic cDNAs were stored at −80 °C until further use as template for real-time qPCR.

### 2.5. Real-Time qPCR Analysis

The primers utilized in this study are listed in Table 1 and were obtained from Integrated DNA Technologies (Coralville, IA, USA). Each qPCR reaction consisted of 10 μL of 2 × PowerUp SYBR Green PCR Master Mix (Applied Biosystems, Waltham, MA, USA), a 10 μL mixture containing 1 μ L of cDNA template, 1 μL forward and reverse primer (0.5 μM), and 8 μL RNase/DNase free water to a final reaction volume of 20 μL. In general, thermocycling conditions for qPCR were as follows: denaturation at 95 °C for 10 min, followed by 40 cycles consisting of 95 °C for 15 s, 60 °C for 1 min; a melting curve analysis from 65 °C to 95 °C was applied after the thermal cycle to confirm the presence of a single amplicon. The reactions were carried out with three duplicates of each sample in a calibrated QS6-Flex real-time PCR detection system (LifeTechnologies, Carlsbad, CA, USA). The standard curves of each gene were generated by a serial dilution of cDNA template from 1:1 to 1:5, 1:25, 1:125, and 1:625. Rainbow trout elongation factor-1 alpha (*ef1a*) was used as a housekeeping gene to normalize the RNA levels of each target gene, and beta-actin (*actb*) was used to confirm the reliability of normalization. Relative expression levels of target genes were calculated by Pfaffl method [25], wherein the arithmetic formula is defined as relative abundance (RA) = (1 + E_target gene_)^DCt^/(1 + E_housekeeping gene_)^ΔΔCt^; ^Δ^Ct = Ct_control_ − Ct_treatment_, where E represents the PCR efficiency (E = (10^−1/sl^°^pe^−1) × 100%) and Ct = the threshold cycle. Genes with RA > 1 are defined as upregulation; RA < 1 are defined as downregulation. The standard errors were determined as STDEV (value of relative abundance)/SQRT (count(number of relative abundance)).

### 2.6. Statistics and Bioinformatics

The significance (*p* < 0.05) of differentially expressed genes (DEGs) was analyzed by two-way analysis of variance (ANOVA) with time (2, 4, or 24 h) and treatment (rt-Ghrl or rt-desVRQ-Ghrl) as independent variables (main effects). Statistically significant differences between groups were defined as ANOVA F-test results having *p* < 0.05 for the fixed effects and interactions. Select pair-wise comparisons were performed on the group means, with Fisher’s LSD (least significant difference) difference test (two-tailed), when ANOVA indicated a significant (*p* < 0.05) difference for fixed effects and/or interactions using Minitab statistical software package (State College, PA, USA, V 19.2.0). Graphs were prepared by Prism 9 (GraphPad Software, San Diego, CA, USA, V 9.2.1). The heatmap was generated by heatmapper web tool (http://www.heatmapper.ca/expression/ (accessed on 27 January 2023)) [26], and the hierarchical clustering analyses were conducted via using average linkage for clustering method, and absolute values of Pearson correlation coefficient (between 0 to 1) were used to compute the distance between genes. Raw data generated can be seen in supplementary material.

## 3. Results

### 3.1. Hierarchical Clustering Analyses of Differentially Expressed Immune Relevant Genes

**Figure 1:**
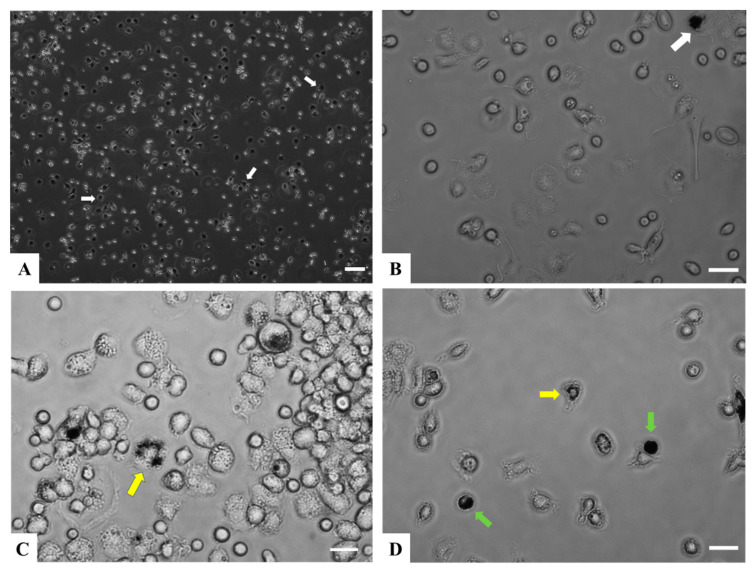
Adherent RT-HKD primary cells under in vitro culture conditions. (**A**) Phase contrast image at 72 h after removal of nonadherent cells at 100×. The white arrows indicate cells showing morphology of macrophage-like cells. (**B**) Image of cells at 72 h after removal of nonadherent cells, 400×. The white arrow indicates a cell showing morphology of macrophage-like cells. (**C**) Phase contrast image of a group of various types of granulocytes at 6 days after removal of nonadherent at 400×. The yellow arrow indicates a multi-lobed (polymorph) nucleus leukocyte. (**D**) Phase contrast image of a group of small lymphoid-like cells at 6 days after removal of nonadherent at 400×. The yellow arrow indicates a multi-lobed nucleus leukocyte, and the green arrows point to cells with morphology consistent with large mononuclear lymphocytes. Scale bar A = 100 μm; B, C, D = 25 μm. Brightness of image (**B**–**D**) was increased post-capture using Adobe Photoshop.

High differentiation of myeloid and lymphoid cells was achieved for ghrelin analogs treatments after RT-HKD primary cell cultures were incubated without interruption for six days post-removal of non-adherent cells (Figure 1). To profile the differential expression levels of immune-relevant genes, RT-HKD cells were left untreated or treated with ghrelin isoforms for 0 h, 2 h, 4 h, and 24 h. Expression of selective genes (Table 1) was measured by reverse transcriptase-coupled qPCR, and normalization was conducted as described in Section 2.

**Figure 2:**
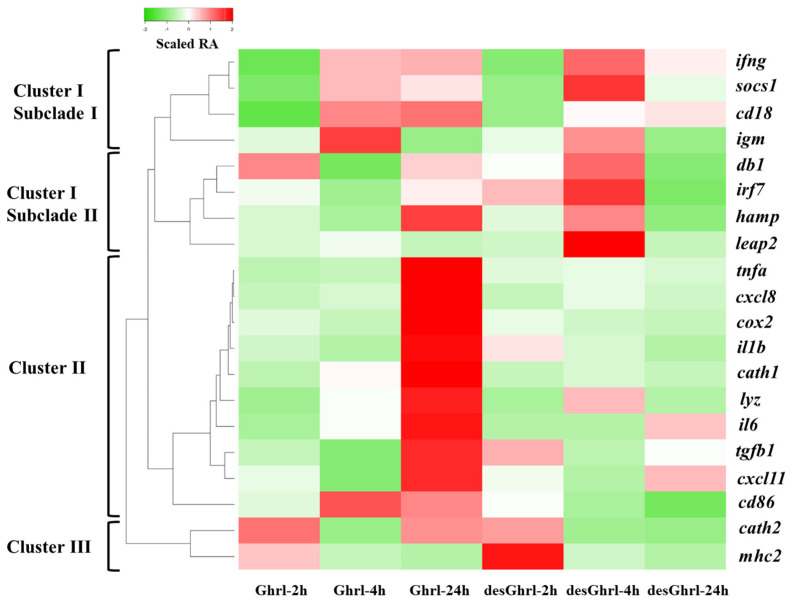
Heatmap with hierarchical clustering analysis of differentially expressed genes over time. A heatmap of immune-relevant genes hierarchically clustered by the expression levels of each mRNA (*n* = 4) × treatment of ghrelin analogs at different time points. Columns show different time points post-treatment of ghrelin analogs, and rows show DEGs. Color gradient denotes the scaled relative abundances, and the clusters were computed by the algorithm of average linkage with the distance measurement equal to the absolute value of Pearson correlation coefficient.

**Figure 3:**
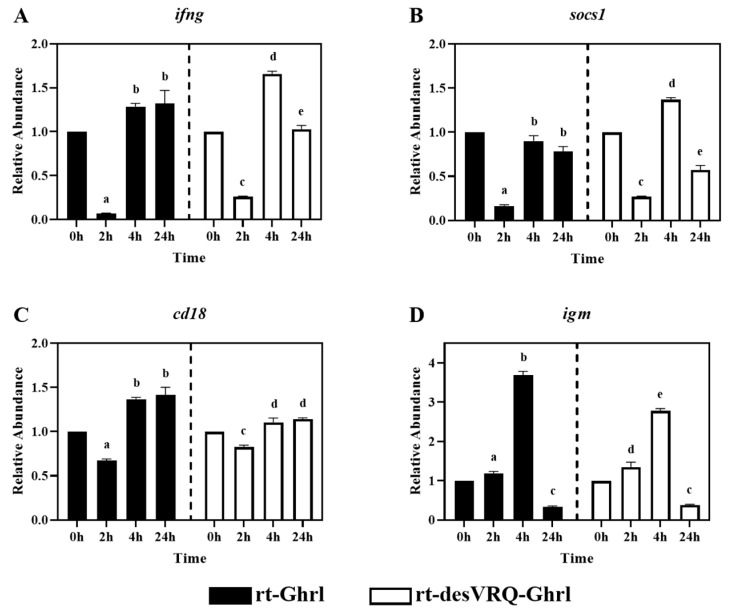
Regulatory effects of ghrelin analogs on relative abundances of genes involved in Cluster I-Subclade I in RT-HKD cells. The differential expression levels of (**A**) interferon-gamma (*ifng*), (**B**) suppressor of cytokine signaling 1 (*socs1*), (**C**) cluster of differentiation 18, integrin b2, (*cd18*), and (**D**) immunoglobulin M (*igm*) were measured by real-time qPCR. The relative abundances (RAs) of each gene of interest were quantified in responding to ghrelin analogs treatment or L15 control. The bars presented the RA of each mRNA as means ± SEM (*n* = 4). The black bars indicate the RA of each mRNA after rt-Ghrl treatment and white bars indicate the RA after rt-desVRQ-Ghrl treatment. Significant differences were analyzed by using two-way ANOVA with time + treatment as main effects. When there was a significant ANOVA effect (*p* < 0.05), differences between conditions were determined by pair-wise comparisons of the group means with Fisher’s LSD test. Alpha scripts represent significant differences (*p* < 0.05) among conditions for each specific gene; groups with the same alpha scripts are not significantly (*p* > 0.05) different from one another.

**Figure 4:**
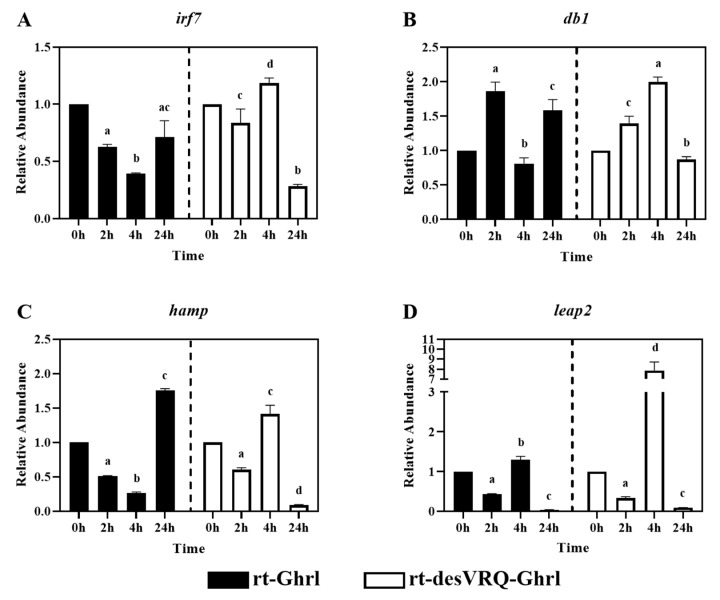
Regulatory effects of ghrelin analogs on relative abundances of genes involved in Cluster I-Subclade II in RT-HKD cells. The differential expression levels of (**A**) interferon regulatory factor 7 (*irf7*), (**B**) beta-defensin (*db1*), (**C**) hepcidin antimicrobial peptide (*hamp*), and (**D**) liver-expressed antimicrobial peptide 2 (*leap2*) were measured by real-time qPCR. The RA of each gene of interest was quantified in responding to ghrelin analogs treatment or L15 control. The bars presented the RAs of each mRNA as means ± SEM (*n* = 4). The black bars indicate the RA of each mRNA after rt-Ghrl treatment and white bars indicate the RA after rt-desVRQ-Ghrl treatment. Significant differences were analyzed by using two-way ANOVA with time + treatment as main effects. When there was a significant ANOVA effect (*p* < 0.05), differences between conditions were determined by pair-wise comparisons of the group means with Fisher’s LSD test. Alpha scripts represent significant differences (*p* < 0.05) among conditions for each specific gene; groups with the same alpha scripts are not significantly (*p* > 0.05) different from one another.

**Figure 5:**
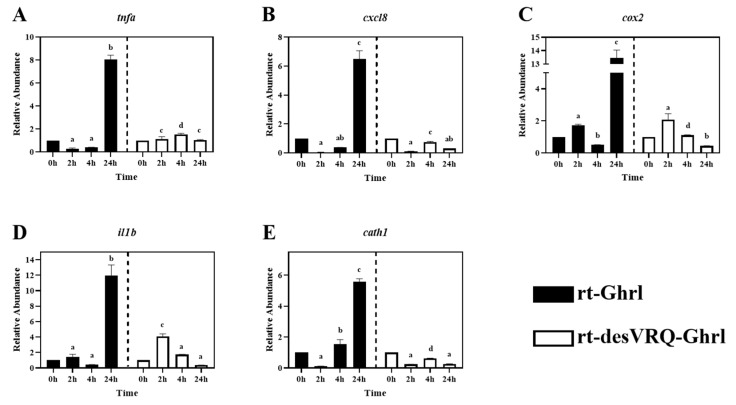
Regulatory effects of ghrelin analogs on relative abundances of genes involved in Cluster II in RT-HKD cells (Part 1). The differential expression levels of (**A**) tumor necrosis factor-alpha (*tnfa*), (**B**) C-X-C motif chemokine ligand 8 (*cxcl8*), (**C**) cyclo-oxygenase 2 (*cox2*), (**D**) interleukin 1 beta (*il1b*), (**E**) cathelicidin I (*cath1*) were measured by real-time qPCR. The RA of each gene of interest was quantified in responding to ghrelin analogs treatment or L15 control. The bars presented the RA of each mRNA as means ± SEM (n = 4). The black bars indicate the RA of each mRNA after rt-Ghrl treatment and white bars indicate the RA after rt-desVRQ-Ghrl treatment. Significant differences were analyzed by using two-way ANOVA with time + treatment as main effects. When there was a significant ANOVA effect (*p* < 0.05), differences between conditions were determined by pair-wise comparisons of the group means with Fisher’s LSD test. Alpha scripts represent significant differences (*p* < 0.05) among conditions for each specific gene; groups with the same alpha scripts are not significantly (*p* > 0.05) different from one another.

**Figure 6:**
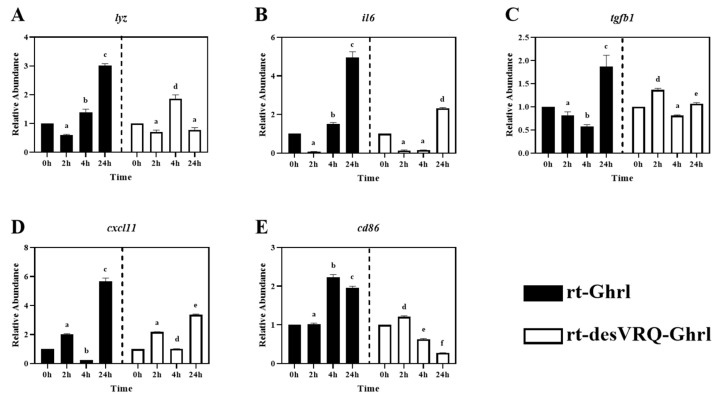
Regulatory effects of ghrelin analogs on relative abundances of genes involved in Cluster II in RT-HKD cells (Part 2). The differential expression level of (**A**) lysozyme (*lyz*), (**B**) interleukin 6 (*il6*), (**C**) transforming growth factor beta 1 (*tgfb1*), (**D**) C-X-C motif chemokine ligand 11 (*cxcl11*), and (**E**) cluster of differentiation 86 (*cd86*) were measured by real-time qPCR. The RAs of each gene of interest were quantified in responding to ghrelin analogs treatment or L15 control. The bars presented the RA of each mRNA as means ± SEM (*n* = 4). The black bars indicate the RA of each mRNA after rt-Ghrl treatment and white bars indicate the RA after rt-desVRQ-Ghrl treatment. Significant differences were analyzed by using two-way ANOVA with time + treatment as main effects. When there was a significant ANOVA effect (*p* < 0.05), differences between conditions were determined by pair-wise comparisons of the group means with Fisher’s LSD test. Alpha scripts represent significant differences (*p* < 0.05) among conditions for each specific gene; groups with the same alpha scripts are not significantly (*p* > 0.05) different from one another.

**Figure 7:**
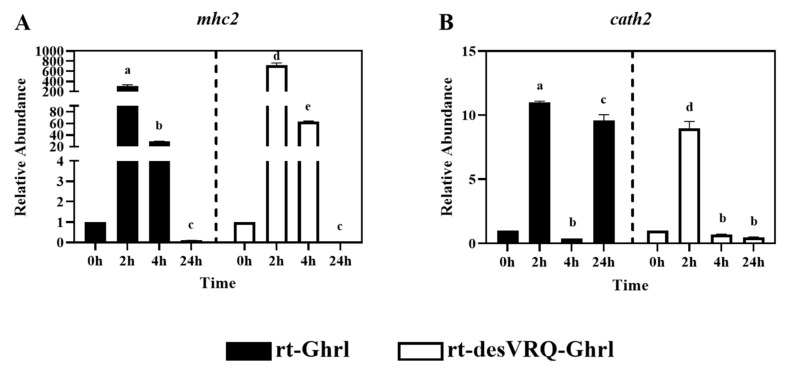
Regulatory effects of ghrelin analogs on relative abundances of genes involved in Cluster III in RT-HKD cells. The differential expression levels of (**A**) major histocompatibility complex class II (*mhc2*) and (**B**) cathelicidin II (*cath2*) were measured by real-time qPCR. The RAs of each gene of interest were quantified in responding to ghrelin analogs treatment or L15 control. The bars presented the RA of mRNA as means ± SEM (*n* = 4). The black bars indicate the RA of each mRNA after rt-Ghrl treatment and white bars indicate the RA after rt-desVRQ-Ghrl treatment. Significant differences were analyzed using two-way ANOVA with time + treatment as main effects. When there was a significant ANOVA effect (*p* < 0.05), differences between conditions were determined by pair-wise comparisons of the group means with Fisher’s LSD test. Alpha scripts represent significant differences (*p* < 0.05) among conditions for each specific gene; groups with the same alpha scripts are not significantly (*p* > 0.05) different from one another.

To visualize the expression pattern of differentially expressed genes (DEGs), a heatmap was generated and the color gradient denotes the change in relative abundance (RA). As shown in Figure 2, the hierarchical clustering analysis was conducted by computed Z-scores, which represent the relationship of normalized and scaled RAs of each gene to the mean of a group of genes, revealing the distances between differentially expressed genes (rows) induced by ghrelin treatments. Three major clusters were formed for DEGs relevant to various immune processes. Cluster I was further partitioned into two predominant subclades that had unique patterns of potential physiological significance (Figure 3 and Figure 4). Cluster II consisted of DEGs that established the most divergent responses between two ghrelin isoforms, particularly at 24 h where rt-Ghrl, but not rt-desVRQ-Ghrl, strongly induced expression over baseline (RA = 1) (Figure 5 and Figure 6). Cluster III consisted of early (2 h to 4 h) response genes that showed the most robust induction with ghrelin isoforms (Figure 7). More detailed descriptions of these patterns are presented in subsequent sections.

### 3.2. Genes in Cluster I Partition into Two Distinct Subclades

As shown in Figure 3, four immune-relevant genes, i.e., interferon-gamma (*ifng*), suppressor of cytokine signaling 1 (*socs1*), integrin b2 (*cd18*), and immunoglobulin M (*igm*), were clustered in subclade I of cluster I. In general, all four genes exhibited similar expression profiles in response to the two ghrelin isoform treatments. In particular, *ifng*, *socs1,* and *cd18* were suppressed in expression at 2 h but exhibited nondifferential or augmented expression levels at 4 and 24 h post-rt-Ghrl treatment. Treating with rt-desVRQ-Ghrl yielded expression profiles that were similar to rt-Ghrl, with suppression at 2 h, overexpression by 4 h, and either sustained induction or return to basal expression level (RA ≅ 1) by 24 h (Figure 3A–C). Although clustered with these other immune-related genes, *igm* exhibited a slightly different pattern, with rt-Ghrl and rt-desVRQ-Ghrl inducing elevated expression levels at 2 h and 4 h but suppressed induction at 24 h (Figure 3D). Statistical analyses revealed that four genes were significantly affected by Treatment (*p* < 0.000), time (*p* < 0.000), and a treatment × time interaction (*p* < 0.000) in ghrelin-treated RT-HKD cells. Additionally, the responses to both ghrelin treatments differed at various time points. The over-time effects of rt-Ghrl treatment were greater than rt-desVRQ-Ghrl on *ifng* (LS means 0.98 vs. 0.89), *cd18* (LS means 1.15 vs. 1.02) and *igm* (LS means 1.74 vs. 1.50) in RT-HKD cells. However, rt-desVRQ-Ghrl treatment showed higher effects on *socs1* (LS means 0.8 vs. 0.6) than rt-Ghrl treatment in RT-HKD cells.

Subclade II of cluster I contained one transcription factor, interferon regulatory factor 7 (*irf7*), and three antimicrobial peptide (AMP) genes, i.e., beta-defensin 1 (*db1*), hepcidin antimicrobial peptide (*hamp*) and liver-expressed antimicrobial peptide 2 (*leap2*). As with subclade I, highly similar over-time effects on expression patterns with three of these four genes, including *irf7*, *db1,* and *hamp* (Figure 4A–C). These genes were inhibited significantly at 4 h but induced at 24 h following rt-Ghrl treatment. In contrast, rt-desVRQ-Ghrl exerted a divergent over-time regulatory profile, expression elevated significantly at 4 h and suppressed, in some cases strongly, at 24 h. Although *leap2* was somewhat distinct from the other genes in its response to rt-Ghrl, it mirrored the aforementioned response to rt-desVRQ-Ghrl, justifying its inclusion in the subclade (Figure 4D). Statistically, three of the genes (*irf7*, *hamp,* and *leap2*) showed significant effects of treatment (*p* < 0.000), time (*p* < 0.000), and a treatment × time interaction (*p* < 0.000). While there was no significant effect of treatment (*p* < 0.99), there were significant effects of time (*p* < 0.000) and a significant treatment × time interaction (*p* < 0.000) on RAs of *db1* mRNA in RT-HKD cells. In general, the main distinguishing feature of this subclade was the inclusion of most of the AMPs tested.

### 3.3. Cluster II: Immune-Associated Genes with Divergent Late-Stage Induction by rt-Ghrl versus rt-desVRQ-Ghrl

As shown in Figure 2, ten genes relevant to different immune activities were associated with cluster II including: (1) cytokines, i.e., tumor necrosis factor-alpha (*tnfa*), interleukin 1 beta (*il1b*), interleukin 6 (*il6*), (2) transforming growth factor 1 beta (*tgfb1*), (3) chemokine ligands, i.e., C-X-C motif chemokine ligand 8 (*cxcl8*) and 11 (*cxcl11*), (4) enzymes, i.e., lysozyme (*lyz*) and cyclo-oxygenase 2 (*cox2*), (5) surface protein, cluster of differentiation 86 (*cd86*), and (6) antimicrobial peptide, cathelicidin 1 (*cath1*). Overall, this was the most homogeneously distinct cluster, with most members exhibiting highly differential gene expression patterns following rt-Ghrl versus rt-desVRQ-Ghrl treatment (Figure 5 and Figure 6). All ten genes exhibited statistically significant induction by rt-Ghrl at 24 h post-treatment, but only two (*il6* and *cxcl11*) induced more than two-fold induction at 24 h by rt-desVRQ-Ghrl. As the largest overall cluster in terms of the number of genes represented, this pattern points to a clear differential regulatory response that likely extends to additional genes of physiological distinction.

Other than *tgfb1*, nine of ten genes in cluster II showed significant effects of treatment (*p* < 0.000), time (*p* < 0.000) and a treatment × time interaction (*p* < 0.000). Over time, the calculated value of LS means indicated that the effects of rt-Ghrl on these eight genes were greater than rt-desVRQ-Ghrl in RT-HKD. Although *tgfb1* was not affected by treatment (*p* = 0.96), there were significant effects of time (*p* < 0.000) and treatment × time interaction (*p* < 0.000). In addition, *tgfb1* mRNA increased through time-course (LS means, 2 h = 0.7, 4 h = 1.1, and 24 h = 1.5), further underscoring the absence of a treatment effect on this mRNA (Figure 6C).

### 3.4. Cluster III Early Response Genes Are Strongly Induced by Both Ghrelin Isoforms

Cathelicidin 2 (*cath2*) and major histocompatibility complex class II (*mhc2*) were partitioned into Cluster III (Figure 2). As shown in Figure 7A, mRNA levels of *mhc2* were massively upregulated at 2 h by treating with both ghrelin isoforms in RT-HKD cells. By 24 h, *mhc2* mRNA had dropped below basal expression level with rt-Ghrl and rt-desVRQ-Ghrl treatments, characteristic of a potently transient, early response gene. For *cath2*, an even more transient induction was observed (Figure 7B), with the exception of the 24 h response to rt-Ghrl, where a strong, possibly secondary response, yielded a re-induction of expression that rivaled the initial response. Statistical analyses showed that RAs of *mhc2* and *cath2* mRNA were significantly affected by treatment (*p* < 0.000), time (*p* < 0.000), and a treatment × time interaction (*p* < 0.000) in RT-HKD cells. In addition, different from genes in cluster I and II, the over-time effects of rt-desVRQ-Ghrl treatment (LS means 1.7 vs. 1.12) on *mhc2* and *cath2* were greater than rt-Ghrl in RT-HKD cells.

## 4. Discussion

### 4.1. Ghrelin Modulates Host Immune Processes via Endocrine Axes and Cytokine Signaling Transduction Pathways

Ghrelin is a multifunctional hormone/cytokine that was initially discovered as a ligand for the growth hormone (GH) secretagogue receptor, which functions to influence central growth hormone release [1]. It is well known to regulate energy homeostasis, satiety, and reproduction, but it also modulates the immune system in many species [1,2,4,5]. In fish, accumulated evidence supports the notion that ghrelin regulates various immune pathways that influence the host immunity [5,10,15,27], which is consistent with the immunomodulatory effects of GH in fish [28]. Given the apparent broad immunomodulatory effects of the GHS and GH system in finfish and the apparent crosstalk between the two, it is possible that ghrelin alters the expression of genes relevant to various immune processes independently of central GH stimulation.

Preliminary studies also suggest that ghrelin generally suppresses pro-inflammation genes in various animal models [4]. However, in fish, in vivo pathogenic challenge and exogenous treatment with GHS analogs have shown divergent results. For instance, in vivo treatment of hybrid tilapia with ghrelin promotes expression level of *il1b* in the liver and kidney after 5 h and 10 h of infection with *Aeromonas hydrophila* and inhibited *tnfa* mRNA in the liver at 10 h and kidney at 5 h [29]. Another study reported that in vivo growth hormone-releasing peptide-6 (Ghrp6) (an agonist of the GHSR that mimics the effect of ghrelin) elevated the expression level of *il1b* mRNA in the head kidney but inhibited it in the spleen of tilapia challenged with *Pseudomonas aeruginosa* [30]. In addition to modulating pro-inflammation, ghrelin was reported to stimulate phagocytosis and superoxide production in rainbow trout leukocytes [19] as well. Other reports revealed the regulatory effects of ghrelin on multi-functional bioregulators such as *ifng*, *tnfa,* and *tgfb1,* etc., which may suggest that ghrelin modulates host immune responses via mediating cytokine/chemokine signaling pathways [4,29,31,32]. In the current study, we evaluated the regulatory effects of ghrelin analogs on cytokines, chemokines, transcription factors, interferon, and growth factor, and the differential expression of these bioregulators may result in functional perturbations of corresponding immune pathways. By combining our findings and the literature evidence, these results indicate ghrelin modulates host immune processes via not only the central regulation of the somatotropic axis but also through cytokine/chemokine signaling transduction pathways.

### 4.2. Primary Cell Culture Represents Head Kidney as an Immune Competent Organ in Rainbow Trout

Primary cells isolated from RT-HKD have been a valuable model to study fish immune processes [24,33]. For instance, stress hormones modulated the productions of *il1b* and *il6* in the head kidney primary cell model of rainbow trout and sea bream (*Sparus aurata*) [34]. Additionally, RT-HKD cells have been employed to study the effects of cytokine stimulation on the differentiation of leukocytes and lymphocytes [35]. To minimize the use of experimental animals and avoid potential complexities associated with in vivo ghrelin treatment, we adapted this compatible in vitro system to explore the direct immunomodulatory effects of ghrelin on RT-HKD primary cells. Several major types of myeloid and lymphoid cells were observed and differentiated to maturation in the living culture system as presented in Figure 1. Such observations affirm that this in vitro model is sufficient to represent at least a portion of the immune-relevant cellular components of the RT-HKD and will further enable evaluation of the direct actions of ghrelin isoforms in this finfish. Expression patterns of twenty immune-relevant genes were profiled by reverse transcriptase-coupled qPCR at three time points after treatment with rainbow trout ghrelin analogs in RT-HKD. Among these DEGs, three major clusters were identified in the hierarchical analysis, differences within Cluster I were significant enough to divide this cluster into two subclades (Figure 2). The modulatory effects will be discussed in the following sections.

### 4.3. Ghrelin Isoforms Exert Differential Modulation of Immune Relevant Genes in Two Distinct Subclades in Cluster I

As mentioned, the differences in RAs were significant enough for us to divide Cluster I into two distinct subclades (Figure 3 and Figure 4). By comparing the expression patterns induced by ghrelin isoforms in subclade I (Figure 3), we observed that both isoforms moderately suppressed genes at 2 h, but that rt-desVRQ-Ghrl inhibited expression at 24 h, while levels returned to basal expression (RA ≅ 1) at 24 h with rt-Ghrl. For DEGs in subclade II (Figure 4), with the exception of *db1* at 2 h, rt-Ghrl inhibited the expression levels at 2 h or 4 h, but rt-desVRQ-Ghrl enhanced these mRNAs at 4 h and suppressed them at 24 h. This conserved divergence in the effects of the two ghrelin analogs indicates that significant physiological responses, and thus biological functions, likely exist in rainbow trout. Furthermore, while being inhibited by rt-Ghrl at 2 or 4 h, the expression levels of *irf7*, *db1*, *hamp,* and *leap2* were promoted by rt-desVRQ-Ghrl at 4 h. This observation may suggest that the two peptides exert dynamic feedback regulation (i.e., secondary effects) at different time points, or that separate signaling pathways are engaged.

Interestingly, besides cathelicidins (*cath1*/*2*), all AMPs evaluated in this study are clustered in subclade II. Many studies indicated that GHS modulates the expressions of various antimicrobial peptides in multiple fish species [20,29]. For example, Ghrp6 (a mimic of GHS) induces the overexpression of piscidin-like AMP in tilapia (*Oreochromis sp.*) [30], while pituitary adenylate cyclase-activating polypeptide, a type of growth hormone releasing hormone, promotes expression of pardaxin and hepcidin in African sharptooth catfish (*Clarias gariepinus*) [36]. In the present study, we demonstrated that ghrelin analogs alter the expression of antimicrobial peptides at different time points and assessed different AMP genes. Many reports showed that the production of AMPs was often regulated by Toll-like receptors (TLRs) and NOD-like receptors (NLRs) mediated NF-κB signaling pathways [37,38], and ghrelin is known to regulate NF-κB signaling in various species [5,18,39]. Therefore, our results may suggest an alternative route where ghrelin isoforms modulate the host innate immune responses via TLRs/NLRs signaling, and the differential expressions of AMPs may result from a combination of ghrelin-mediated functional perturbations of GHS and NF-κB signaling pathways in RT-HKD cells.

### 4.4. Ghrelin Isoforms Modulate Divergent Late-Stage Responses on DEGs in Cluster II

All of the DEGs assigned to Cluster II via hierarchical analysis were significantly induced by rt-Ghrl treatment at 24 h, but, with some exceptions, either returned to baseline or were slightly inhibited by rt-desVRQ-Ghrl at 24 h (Figure 5 and Figure 6). This finding affirms the notion that two ghrelin isoforms play divergent roles in modulating host immunity at various time points. Many cytokines, chemokines, and transcription factors, e.g., *il1b*, *il6*, *cxcl8*, *cxcl11*, *tnfa*, and *tgfb1*, were found in this cluster. The lateness of the induction (24 h) in response to rt-Ghrl is notable and may suggest a delayed or even a secondary induction. Because induction of these same genes by rt-desVRQ-Ghrl either was not observed or was less significant, the types of signaling cascades induced must surely differ. If the pattern is due to crosstalk among different signaling pathways, it seems that clarifying which pathways are differentially induced will assist significantly in our efforts to understand the physiological distinctions in these isoforms. Alternatively, if secondary pathways regulate cytokine/chemokine signaling, then identifying the early responders (i.e., those falling into cluster III) will help to predict which intermediates mediate cytokine/chemokine signaling cascades that may be distinct from those activated by rt-Ghrl analogs directly [22,40,41,42]. Furthermore, multi-functional bioregulators (*ifng*, *tnfa,* and *tgfb1*) showed similar expression patterns, inhibited at early and enhanced at late stages, by rt-Ghrl treatment. Since these regulators mediated the production of many cytokines and chemokines [4,40,43], the perturbed downstream signaling of these bioregulators can alter the expression of cytokines and chemokines. Therefore, the combining effect from *ifng*, *tnfa,* and *tgfb1* may also impact the secondary, or even tertiary responses.

As mentioned in 4.1, evidence indicated that ghrelin and GHS modulate inflammation via altering cytokine signaling pathways in teleost fish [29,30,36]. By combining this notion and our observations of significant secondary inductions of pro-inflammation cytokines (*tnfa*, *il1b,* and *il6*), and enzyme (*cox2*), these studies suggest that rt-Ghrl promotes expressions of pro-inflammation genes at late-stage and, thus, may exhibit pro-inflammation activity in RT-HKD cells, whereas rt-desVRQ-Ghrl does not exert similar regulation. In addition, a common upregulation of *ifng* and *tgfb1* mRNAs at 24 h were induced by rt-Ghrl (rather than rt-desVRQ-Ghrl) in RT-HKD cells (Figure 3A and Figure 6C). Since *tgfb1* induces expressions of *il1b* and *il6* in lymphocytes [31,42] and *ifng* mediates the production of various types of cytokines in immune-relevant cells [22,40], our findings may suggest that ghrelin modulates inflammation via two routes: directly mediating cytokine expression and indirectly altering downstream signaling of multi-functional bioregulators (*tnfa*, *ifng,* and *tgfb1*), simultaneously.

### 4.5. Ghrelin Isoforms Induce Strong Early Responses on DEGs in Cluster III

The overexpression of *mhc2* and *cath2* was strongly induced by both ghrelin peptides at an early stage (2 h), and the inductions dropped over time and finally below to basal expression level at 24 h. The strong induction of *mhc2* is reminiscent of an immediate-early cytokine response, such as *ifng* responses that elicit rapid, transient upregulation of *mhc2* in most cells via JAK/STAT signaling—a pathway anticipated to modulate indirect ghrelin effects [28,40,44]. Additionally, *cd86* encodes a constitutive expression co-stimulator protein (B7-2) on antigen-presenting cells (APCs) and mediates cell–cell association during antigen presenting process [45,46]. By combining the ghrelin-induced overexpression of *mhc2* (antigen binding molecule) and *cd86* (stimulator of APCs) at an early stage (2–4 h), this finding may suggest ghrelin modulates the enhancement of antigen-presenting process early, but negative feedback regulates this process at a late stage in RT-HKD cells.

Although *cath1* and *cath2* showed different patterns, some similarities of late-stage (24 h) inductions were observed in their expression profiles. As shown in Figure 5E and Figure 7B, rt-Ghrl promoted late inductions of both *cath1* and *cath2* at 24 h, whereas rt-desVRQ-Ghrl inhibited the expressions of both cathelicidin paralogs at this late stage. This finding once again suggests that divergent actions of ghrelin analogs on different assessed genes may lead to differentially expressed *cath1* and *cath2* at 24 h, but that changes to related gene targets such as these may be subtle and change only the magnitude or timing of the response.

## 5. Conclusions

Using an in vitro system, we demonstrated that two ghrelin isoforms differentially modulate immune-related genes in rainbow trout cells. Our results support the hypothesis that ghrelin modulates host immunity via not only the somatotropic axis but perhaps also via cytokine signaling pathways—some activated directly, some responding indirectly. The divergent expression patterns of the responsive genes indicate that the two ghrelin peptides exert different functional perturbations in regulating immune-relevant genes in primary RT-HKD cells. These differing actions suggest the two analogs may activate unique pathways and, therefore, elicit distinct responses in fish immunity. Although we have directly assessed the immunomodulatory effects of ghrelin isoforms in RT-HKD cells, further studies will be required to identify the full cohorts of responsive genes and to clarify the differential roles and mechanisms by which these ghrelin analogs affect immunity in rainbow trout.

## Figures and Tables

**Table 1:** Primer sequences of genes relevant to immune processes.

Name	Target Gene	Forward (5′-3′)	Reverse (5′-3′)	Amplicon	RefSeq
*actb*	Beta actin	CCTTCCTCGGTATGGAGTCTT	ACAGCACCGTGTTGGCGTACAG	105	NM_001124235.1
*ef1a*	Elongation factor 1 alpha	GATCCAGAAGGAGGTCACCA	TTACGTTCGACCTTCCATCC	150	NM_001124339.1
*il1b*	Interleukin 1 beta	ACATTGCCAACCTCATCATCG	TTGAGCAGGTCCTTGTCCTTG	91	NM_001124347.2
*il6*	Interleukin 6	ACTCCCCTCTGTCACACACC	GGCAGACAGGTCCTCCACTA	91	NM_001124657.1
*cxcl8*	C-X-C motif chemokine ligand 8	CGCTGCATTGAGACGGAAAG	AGCGCTGACATCCAGACAAA	144	NM_001124362.1
*tnfa*	Tumor necrosis factor alpha	GGGGACAAACTGTGGACTGA	GAAGTTCTTGCCCTGCTCTG	66	NM_001124374.1
*irf7*	Interferon Regulatory Factor 7	AGCAATACACTGGTTTGTTC	GTGGGATGCTCATTGATTTTC	145	XM_021600499.2
*ifng*	Interferon-gamma	GGAGCCACAGTTGAGATACATA	ACCATTGAGTATTGTGCGTGAT	124	NM_001124620.1
*cd18*	Integrin beta 2	GGAGTTCCGCAGGTTTGAGA	GAAGGTAGGGTTAGCCACGG	103	NM_001124435.1
*cd86*	Cluster of differentiation 86	ATCCCCTCACCCGGAGTATT	GCATTCGTACAGCCCCTCAT	103	NM_001160477.1
*cxcl11*	C-X-C motif chemokine ligand 11	ATTCACGATGGCCTTCGCAC	CATCTGGCACCTGGAACGTGT	105	XM_021556956.2
*mhc2*	Major histocompatibility complex class II	ATCTCAGATTCAACAGCACTGTGGG	CGTTAGGCTTACATAGACGCTCCAG	135	NM_001195534.1
*cox2*	Cyclooxygenase 2	CCAGTACCAGAACCGTATCGCAG	GTCCACCAGCCACCCTTCC	200	XM_021611217.2
*lyz*	Lysozyme	GAAACAGCCTGCCCAACT	GTCCAACACCACACGCTT	239	XM_021601582.2
*tgfb1*	Transforming growth factor beta 1	CATGTCCATCCCCCAGAACT	GGACAACTGTTCCACCTTGTGTT	361	XM_036961610.1
*socs1*	Suppressor of cytokine signaling 1	GATTAATACCGCTGGGATTCTGTG	CTCTCCCATCGCTACACAGTTCC	136	NM_001146166.1
*hamp*	Hepcidin	GGTCGTCCTCGCATGTATGTT	TCGCACCTCGGAGAAAGGAT	61	XM_036964755.1
*db1*	Beta defensin	GGATTCTTGTGCTGTGTTTCTCAT	ACATGCGGATGTGATGTCTTCA	95	NM_001124434.1
*leap2*	Liver-expressed antimicrobial peptide 2	GGAGAAGCCTTTCTGGTTTCAG	GTCCAAAAGCTAGCATTCAATATCAC	130	NM_001124464.1
*cath1*	Cathelicidin 1	TCACAGTGATGGAAATCGC	CATTTCTTTGATAGATGGGTTCT	83	NM_001124480.1
*cath2*	Cathelicidin 2	TGGGTAGAAAAGATTCCAAGG	GCTAGCTCCAGCAATGCC	89	NM_001124463.1

## Data Availability

Raw data were generated at USDA-ARS in School of Freshwater Sciences of University of Wisconsin in Milwaukee, and were uploaded as Supplemental File S1. Derived data supporting the finding of this study are available from the corresponding author, Yueh-Chiang Han, on proper request.

## Supplementary Materials

The following supporting information can be downloaded at: https://www.mdpi.com/article/10.3390/ani13101683/s1.

## Abbreviations

Actb:	Beta-actin
ANOVA:	Analysis of variance
AMP:	Antimicrobial peptide
APC:	Antigen-presenting cell
Cath1:	Cathelicidin 1
Cath2:	Cathelicidin 2
Cd18:	Cluster of differentiation 18
Cd86:	Cluster of differentiation 86
Cox2:	Cyclo-oxygenase 2
Ct:	Threshold cycle
Cxcl11:	CXC Motif Chemokine Ligand 11
Cxcl8:	CXC Motif Chemokine Ligand 8
Db1:	Beta-defensin 1 antimicrobial peptide
DEG:	Differential expressed gene
DMEM:	Dulbecco’s Modified Eagle Medium
Ef1a:	Elongation factor-1 alpha
GH:	Growth hormone
rt-Ghrl:	Rainbow trout ghrelin
rt-desVRQ-Ghrl:	Rainbow trout desVRQ ghrelin
GHSR:	Growth hormone secretagogue receptor
GOI:	Gene of interest
Hamp:	Hepcidin antimicrobial peptide
Ifng:	Interferon-gamma
Igm:	Immunoglobulin M
Il1b:	Interleukin-1 beta
Il6:	Interleukin-6
Irf7:	Interferon regulatory factor 7
LEAP2:	Liver-expressed antimicrobial peptide 2
LSD:	Least significant difference
Lyz:	Lysozyme
Mhc2:	Major histocompatibility complex class II
NF-κB:	Nuclear factor kappa b
NLR:	NOD-like receptor
PBS:	Phosphate-buffered saline
RT-HKD:	Rainbow trout head kidney
qPCR:	Quantitative polymerase chain reaction
RA:	Relative abundance
Socs1:	Suppressor of cytokine signaling 1
Tgfb1:	Transforming growth factor beta 1
TLR:	Toll-like receptor
Tnfa:	Tumor necrosis factor alpha
